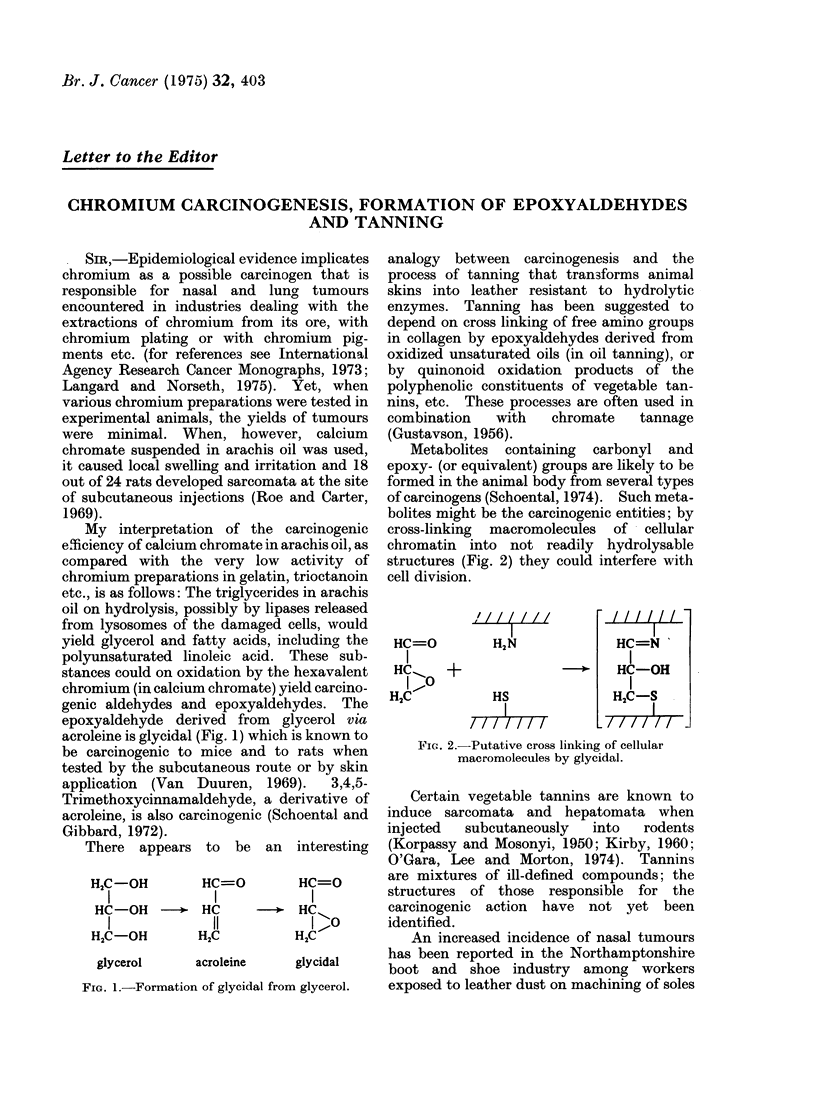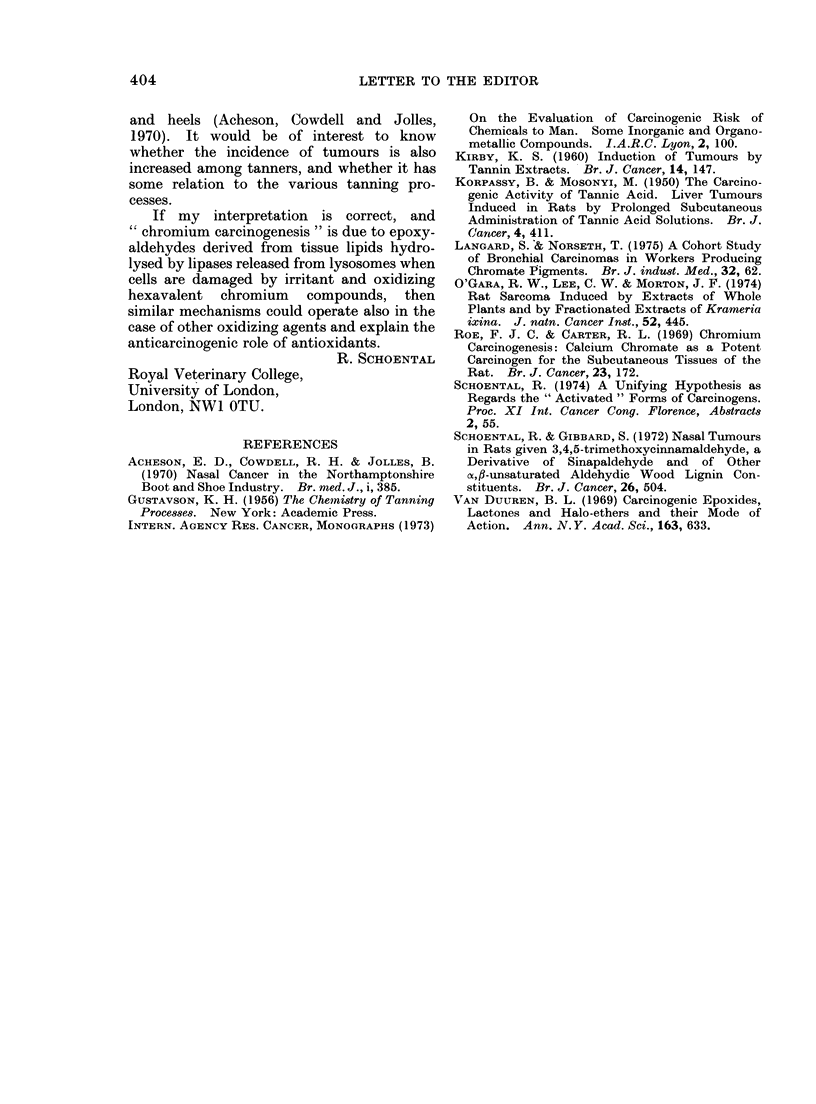# Chromium carcinogenesis, formation of epoxyaldehydes and tanning.

**DOI:** 10.1038/bjc.1975.241

**Published:** 1975-09

**Authors:** R. Schoental


*Br. J. Cancer* (1975) **32**, 403

### *Letter to the Editor*

### CHROMIUM CARCINOGENESIS, FORMATION OF EPOXYALDEHYDES AND TANNING

SIR,—Epidemiological evidence implicates chromium as a possible carcinogen that is responsible for nasal and lung tumours encountered in industries dealing with the extractions of chromium from its ore, with chromium plating or with chromium pigments etc. (for references see International Agency Research Cancer Monographs, 1973; Langard and Norseth, 1975). Yet, when various chromium preparations were tested in experimental animals, the yields of tumours were minimal. When, however, calcium chromate suspended in arachis oil was used, it caused local swelling and irritation and 18 out of 24 rats developed sarcomata at the site of subcutaneous injections (Roe and Carter, 1969).

My interpretation of the carcinogenic efficiency of calcium chromate in arachis oil, as compared with the very low activity of chromium preparations in gelatin, trioctanoin etc., is as follows: The triglycerides in arachis oil on hydrolysis, possibly by lipases released from lysosomes of the damaged cells, would yield glycerol and fatty acids, including the polyunsaturated linoleic acid. These substances could on oxidation by the hexavalent chromium (in calcium chromate) yield carcinogenic aldehydes and epoxyaldehydes. The epoxyaldehyde derived from glycerol *via* acroleine is glycidal (Fig. 1) which is known to be carcinogenic to mice and to rats when tested by the subcutaneous route or by skin application (Van Duuren, 1969). 3,4,5-Trimethoxycinnamaldehyde, a derivative of acroleine, is also carcinogenic (Schoental and Gibbard, 1972).

There appears to be an interesting

FIG. 1.—Formation of glycidal from glycerol.

analogy between carcinogenesis and the process of tanning that transforms animal skins into leather resistant to hydrolytic enzymes. Tanning has been suggested to depend on cross linking of free amino groups in collagen by epoxyaldehydes derived from oxidized unsaturated oils (in oil tanning), or by quinonoid oxidation products of the polyphenolic constituents of vegetable tannins, etc. These processes are often used in combination with chromate tannage (Gustavson, 1956).

Metabolites containing carbonyl and epoxy- (or equivalent) groups are likely to be formed in the animal body from several types of carcinogens (Schoental, 1974). Such metabolites might be the carcinogenic entities; by cross-linking macromolecules of cellular chromatin into not readily hydrolysable structures (Fig. 2) they could interfere with cell division.

FIG. 2.—Putative cross linking of cellular macromolecules by glycidal.

Certain vegetable tannins are known to induce sarcomata and hepatomata when injected subcutaneously into rodents (Korpassy and Mosonyi, 1950; Kirby, 1960; O'Gara, Lee and Morton, 1974). Tannins are mixtures of ill-defined compounds; the structures of those responsible for the carcinogenic action have not yet been identified.

An increased incidence of nasal tumours has been reported in the Northamptonshire boot and shoe industry among workers exposed to leather dust on machining of soles and heels (Acheson, Cowdell and Jolles, 1970). It would be of interest to know whether the incidence of tumours is also increased among tanners, and whether it has some relation to the various tanning processes.

If my interpretation is correct, and " chromium carcinogenesis " is due to epoxy-aldehydes derived from tissue lipids hydrolysed by lipases released from lysosomes when cells are damaged by irritant and oxidizing hexavalent chromium compounds, then similar mechanisms could operate also in the case of other oxidizing agents and explain the anticarcinogenic role of antioxidants.

R. SCHOENTAL

Royal Veterinary College,
University of London,
London, NW1 0TU.

### REFERENCES

ACHESON, E. D., COWDELL, R. H. & JOLLES, B. (1970) Nasal Cancer in the Northamptonshire Boot and Shoe Industry. *Br. med. J.*, i, 385.

GUSTAVSON, K. H. (1956) *The Chemistry of Tanning Processes.* New York: Academic Press.

INTERN. AGENCY RES. CANCER, MONOGRAPHS (1973) On the Evaluation of Carcinogenic Risk of Chemicals to Man. Some Inorganic and Organometallic Compounds. *I.A.R.C. Lyon*, **2**, 100.

KIRBY, K. S. (1960) Induction of Tumours by Tannin Extracts. *Br. J. Cancer*, **14**, 147.

KORPASSY, B. & MOSONYI, M. (1950) The Carcinogenic Activity of Tannic Acid. Liver Tumours Induced in Rats by Prolonged Subcutaneous Administration of Tannic Acid Solutions. *Br. J. Cancer*, **4**, 411.

LANGARD, S. & NORSETH, T. (1975) A Cohort Study of Bronchial Carcinomas in Workers Producing Chromate Pigments. *Br. J. indust. Med.*, **32**, 62.

O'GARA, R. W., LEE, C. W. & MORTON, J. F. (1974) Rat Sarcoma Induced by Extracts of Whole Plants and by Fractionated Extracts of *Krameria ixina*. *J. natn. Cancer Inst.*, **52**, 445.

ROE, F. J. C. & CARTER, R. L. (1969) Chromium Carcinogenesis: Calcium Chromate as a Potent Carcinogen for the Subcutaneous Tissues of the Rat. *Br. J. Cancer*, **23**, 172.

SCHOENTAL, R. (1974) A Unifying Hypothesis as Regards the " Activated " Forms of Carcinogens. *Proc. XI Int. Cancer Cong. Florence, Abstracts* **2**, 55.

SCHOENTAL, R. & GIBBARD, S. (1972) Nasal Tumours in Rats given 3,4,5-trimethoxycinnamaldehyde, a Derivative of Sinapaldehyde and of Other $\alpha,\beta$-unsaturated Aldehydic Wood Lignin Constituents. *Br. J. Cancer*, **26**, 504.

VAN DUUREN, B. L. (1969) Carcinogenic Epoxides, Lactones and Halo-ethers and their Mode of Action. *Ann. N.Y. Acad. Sci.*, **163**, 633.